# No secret hiding place on the ocular surface: what about after systemic SARS-CoV-2 infection?

**DOI:** 10.1007/s00417-021-05230-z

**Published:** 2021-05-18

**Authors:** Alexander C. Rokohl, Gerd Fätkenheuer, Claus Cursiefen, Ludwig M. Heindl

**Affiliations:** 1 Department of Ophthalmology, Faculty of Medicine, University Hospital of Cologne, University of Cologne, Kerpener Straße 62, 50937 Cologne, Germany; 2 Department I of Internal Medicine, Division of Infectious Diseases, University of Cologne, Cologne, Germany; 3 German Center for Infection Research (DZIF), Partner Site Bonn-Cologne, Cologne, Germany; 4 Center for Molecular Medicine Cologne (CMMC), University of Cologne, Cologne, Germany

Dear Editor,

In our prospective study, we looked deeply into the possibility that SARS-CoV-2 may hide on the ocular surface of pre-screened asymptomatic patients in a tertiary eye care center [1]. The risk for an isolated conjunctival viral activity in patients with a negative nasopharyngeal swab-based RT-PCR seems to be absent or extremely low, suggesting no need to perform additional conjunctival swabs in patients with negative nasopharyngeal swabs [1].

However, previous studies reported the presence of the entrance receptor of SARS-CoV-2, the angiotensin-converting enzyme 2 (ACE 2), on the ocular surface [2–4]. In addition, SARS-CoV-2 was detected in ocular tissues, tears, and conjunctival secretions of patients with systemic SARS-CoV-2 infection [2–4]. Therefore, a potential hiding place on the ocular surface after systemic SARS-CoV-2 infection seems to be possible, especially in patients showing unspecific conjunctivitis during COVID-19 or in patients with ongoing systemic symptoms [4–9]. Furthermore, there is not much known about SARS-CoV-2 on the ocular surface in asymptomatic carriers. These potential hiding places might be a significant risk factor of SARS-CoV-2 transmission to both medical staff as well as other patients [10, 11].

In addition, the authors raise the very interesting, yet unanswered and clinically highly relevant question of whether SARS-CoV-2 may—as it is known for Ebola virus [12]—persist inside the immune-privileged eye even if the patient is otherwise healthy and PCR “negative.” This would be compatible with ocular immune privilege [13] and put ophthalmic surgeons and theater personal at risk if ocular surgery is performed even in extraocular “negative” COVID survivors.

Therefore, we fully agree with our highly appreciated colleagues that research regarding other hiding places of SARS-CoV-2 within the eye and on the ocular surface should be expanded. The key question of whether long(er) term SARS-CoV-2 persistence on the ocular surface and intraocularly of COVID-19 survivors or asymptomatic carriers may hold long-term risks is empirically open. Exploring these concerns using targeted research is now a high priority.
